# Nicotine dependence affects cardiopulmonary endurance and physical activity in college students in Henan, China

**DOI:** 10.18332/tid/166133

**Published:** 2023-06-23

**Authors:** Yugang Guo

**Affiliations:** 1 School of Physical Education, Anyang Normal University, Anyang, China

**Keywords:** nicotine dependence, cardiopulmonary endurance, physical activity, VO_2_max, Cigarette Dependence Scale-5 (CDS-5)

## Abstract

**INTRODUCTION:**

This study aimed to investigate the impact of smoking on physical activity level, emotional status, and cardiopulmonary endurance in healthy young Chinese college students in order to develop future nicotine dependence management solutions.

**METHODS:**

This survey-based study was conducted in college students aged 19–26 years who were currently smoking. Cardio-respiratory endurance was assessed by estimating VO_2_max. Participants were given a questionnaire containing five factors from the Cigarette Dependence Scale-5 (CDS-5), also assessed were variables for physical activity level, using the Global Physical Activity Questionnaire (GPAQ), and emotional status. The sports training behavior was assessed using the Coaching Behavior Scale for Sport (CBS-S).

**RESULTS:**

A total of 400 participants were randomly selected and included in the study. All of them were current smokers. The highest percentage of participants had a score of 4 on the CDS-5 (n=93, 23.2%), scored 3–5 on each module of sports training, and experienced negative emotions, particularly depression (n=172; 43.0%) and anger (n=162; 40.5%). VO_2_max levels were significantly lower in participants with high nicotine dependence (CDS-5 score 4–5), and they correlated negatively with CDS-5 scores (r= -0.883, p<0.001). Nicotine dependence scores were negatively correlated with physical activity levels (r= -0.830, p<0.001), and high nicotine dependence scores were independently related to low physical activity (adjusted odds ratio, AOR=14.66; 95% CI: 4.98–43.19 , p <0.001).

**CONCLUSIONS:**

Tobacco smoking has a negative impact on emotional status. It also reduces cardiopulmonary endurance by reducing VO_2_max levels and negatively affects physical activity. Accordingly, it is critical to implement effective tobacco prevention programs for college students, such as a smoking warning system and physical exercise training, as well as to educate them on how to quit smoking.

## INTRODUCTION

Nicotine is a highly addictive substance that enters the lungs through smoking. Cigarette smoking remains the leading avoidable cause of disease and death worldwide. In 2020, 22.3% of the global population, 36.7% of men, and 7.8% of women used tobacco^1^. Approximately half of all smokers die prematurely due to smoking-related diseases. Each year, smoking kills over 8 million people, with approximately 1.2 million deaths caused by secondhand smoke^2^. Therefore, many countries have increased tobacco taxes and enacted stringent legislation prohibiting smoking in stores and public places in order to limit smoking. Smoking addiction is influenced by a number of factors, including age of initiation, negative affective states, mood disorders, psychiatric disorders, and executive functioning^3^.

Evidence suggests that nicotine affects brain development and function, as well as cognitive function. According to a population-based study involving 2163 smokers who had difficulties with their attention, working memory, and ability to regulate their motivation, smoking has a negative impact on cognitive abilities^4^. Furthermore, tobacco use and nicotine dependence are linked to mental disorders such as impaired oral and visuo-spatial learning, mental efficiency, visuospatial recollection, and general knowledge, as well as a number of psychiatric disorders such as suicide ideation, major depressive disorder, and bipolar disorder, with a dose–response relationship between smoking and the risk of suicide and schizophrenia^5,6^.

In addition, tobacco smoking is a significant risk factor for cardiovascular and respiratory diseases. It reduces maximal oxygen consumption (VO_2_max) and has a negative impact on cardiopulmonary endurance. It can cause immune dysfunction, increasing the risk of infectious diseases^7^.

Physical inactivity is the fourth leading cause of premature mortality, accounting for approximately 1.6 million deaths from non-communicable diseases (NCDs) each year^8^. Tobacco use has been linked to physical inactivity, and several epidemiologic studies have found that smoking can affect some behavioral risk factors for chronic diseases, such as physical activity and diet, with an inverse relationship between cigarette use and physical activity in adults^9^. Physical inactivity and smoking are among the leading preventable causes of morbidity and mortality worldwide, and each is associated with a significantly increased risk of a variety of chronic diseases, including chronic lung diseases, coronary heart disease, and cancer, as well as psychiatric disorders like depression, which impose a marked economic burden. Physical activity not only improves overall health and longevity and helps prevent NCDs, but it may also help reduce cigarette cravings and withdrawal symptoms, resulting in better smoking behavior^10^.

Despite significant efforts made by countries in response to the World Health Organization (WHO) Global Action Plan on NCDs to implement regional plans to enhance physical activity, there has been no significant increase in physical activity levels across the population^5^. Furthermore, only a few studies have investigated the impact of smoking on emotional well-being and cardiopulmonary endurance, as well as the bidirectional effect on physical activity^9^. Therefore, the present study aimed to investigate the impact of smoking on physical activity level, emotional status, and cardiopulmonary endurance in healthy young Chinese college students, all of which could affect general health.

## METHODS

### Study design

This survey-based study was conducted from April to August 2022 among randomly selected students from Anyang Normal University and Anyang Institute of Technology in Henan, China, via simple random sampling using the online Random Number Generator (https://www.calculator.net/random-number-generator.html).

The questionnaire was mainly generated online using the online survey platform SurveyMonkey (https://www.surveymonkey.com/) and distributed via links to the students emails, but there was also an offline option to contact people and obtain their responses. The questionnaire was designed to collect information regarding nicotine dependence, physical activity level, emotional status, and cardiorespiratory endurance of the participants. Informed consent was obtained from all subjects involved in the study.

### Inclusion and exclusion criteria

In this study, students from Anyang Normal University and Anyang Institute of Technology in Henan, China, were included. Participants who were willing to answer all questions, were current smokers, aged 19–26 years, gave the required consent, and performed the required assessment for VO_2_max estimation, were included. Participants who did not respond to all questions, as well as those who had chronic cardiorespiratory or psychological disorders as assessed from their medical records, were excluded from the study. After applying inclusion and exclusion criteria, 400 participants were finally included.

### Assessment of dependent factors and outcomes

The degree of tobacco dependence was assessed using the Cigarette Dependence Scale-5 (CDS-5) (Supplementary file Table S1), which consists of five questions assessing tobacco dependence and focusing on various ways of defining influences, excluding tolerance^11^. The physical activity level was determined based on the frequency, time, intensity, and type of physical activity (work, home, and recreational activities) using the Global Physical Activity Questionnaire (GPAQ)^12^. The intensity of physical activity was classified as vigorous (e.g. jogging and cycling), moderate (e.g. gardening and walking at a moderate pace), or mild (e.g. laundry and home repairs). For the analysis of activity level, physical activity was divided into three categories: high physical activity (a minimum of 3 days of vigorous-intensity activities in a week, or at least 7 days of walking, moderate- or vigorous-intensity activities, MVPA, per week), moderate physical activity (not meeting the criteria for high levels, but either having at least 3 days of vigorous-intensity activity for 20 min or more per day, or at least 5 days of moderate intensity activity, or walking for 30 min or more per day, or at least 5 days of combined walking and MVPA per week) and low physical activity (not meeting the criteria for either high or moderate physical activity level)^13^. This measure was developed by WHO and has shown acceptable validity and reliability in determining physical activity levels in various studies and populations^14^.

The cardiopulmonary endurance was determined by estimating VO_2_max using a treadmill test of the participants to assess maximum heart rate (HRmax), and then using the following formula^15^:

VO_2_max = 15 × (HRmax/HRrest)

where HRrest is the resting heart rate. The sports training behavior, including physical training, technical skill, mental preparation, strategy-making, and the capability to set goals, was assessed. The participants underwent various sporting activities according to the Coaching Behavior Scale for Sport (CBS-S), and a score was assigned to each on a scale 0–7 to determine sports training behavior^16^. The number of participants for each score for each training or module was recorded. The emotional status was determined by asking each participant to mark the emotional factors (such as psychological mood) that they thought were present at the time of the survey. The collected data were entered into a database and analyzed using software.

Data on sociodemographic and clinical characteristics, including age, gender, body weight, body mass index, and alcohol consumption, were collected from the students’ college records.

### Statistical analysis

The dataset was analyzed using the Statistical Package for the Social Sciences (SPSS) software version 25 (Armonk, NY, United States: IBM Corp.). The graphs were created with GraphPad Prism version 18 (San Diego, CA 92108, USA). All data were checked for normality using the Kolmogorov-Smirnov test, and all data were found to be non-normally distributed. The numerical data are presented as median and interquartile range (IQR) and compared among groups using the Mann-Whitney U test and Kruskal-Wallis test. The categorical data are presented as frequencies and percentages, and compared among groups using the chi-squared test. The CDS-5, age, physical activity level, and VO_2_max were correlated using Spearman’s correlation analysis and then stepwise linear regression analysis. Univariable and multivariable logistic regression analysis was used to assess the independent covariates related to low physical activity. To determine whether nicotine’s effect on physical activity level was mediated via alteration of cardiopulmonary endurance (VO_2_max), a mediation analysis was performed using the Sobel test. All p-values were two-tailed, and p<0.05 was considered statistically significant.

## RESULTS

### Characteristics of the studied cohort

In this study, 596 participants were screened, and 196 were excluded because they had chronic cardiorespiratory (n=12) or psychological (n=18) disorders or because they did not complete the questionnaire (n=72). In addition, there were 94 non-respondents, with a response rate of 80.1% (79.6% for online vs 83.9% for offline). Finally, 400 participants (Supplementary file Figure S1) were included in the study, mean age of 23.9 ± 2.7 years, with 194 males (48.5%) and 206 females (51.5%). All were current smokers, and 38.8% consumed alcohol. Their median body weight was 59.0 kg (IQR: 12.0), and their median BMI was 21.7 kg/m^2^ (IQR: 4.6). The largest number of participants (n=93; 23.2%) scored 4 on the nicotine dependence CDS-5 (Table 1). Male participants had significantly higher body weight and BMI, as well as a higher incidence of alcohol consumption than female participants, with no significant differences in age or nicotine dependence between males and females (Table 1).

**Table 1:** The characteristics of student participants stratified by gender in terms of tobacco dependence, physical activity, emotional status, and cardiopulmonary endurance, Anyang Normal University and Anyang Institute of Technology, Henan, China, April–August 2022 (N=400)

*Characteristics*	*All (N=400) n (%)*	*Male (N=194) n (%)*	*Female (N=206) n (%)*	*p*
**Age** (years), median (IQR)	24.0 (4.0)	25.0 (4.0)	24.5 (8.0)	0.192
**BMI** (kg/m^2^), median (IQR)	21.7 (4.6)	22.9 (4.4)	20.9 (4.8)	<0.001*
**Body weight** (kg), median (IQR)	59.0 (12.0)	63.0 (12.0)	53.0 (9.0)	<0.001*
**Alcohol consumption**	155 (38.8)	90 (46.4)	65 (31.6)	0.002*
**CDS-5**				
1	74 (18.5)	30 (15.5)	44 (21.4)	0.280
2	75 (18.8)	36 (18.6)	39 (18.9)	
3	76 (19.0)	34 (17.5)	42 (20.4)	
4	93 (23.2)	53 (27.3)	40 (19.4)	
5	82 (20.5)	41 (21.1)	41 (19.9)	
**Physical activity level**				
Low	133 (33.2)	71 (36.6)	62 (30.1)	0.383
Moderate	140 (35.0)	64 (33.0)	76 (36.9)	
High	127 (31.8)	59 (30.4)	68 (33.0)	
**VO_2_max**, median (IQR)	27.0 (11.0)	26.0 (9.0)	28.0 (11.0)	0.010*
**METs**, median (IQR)	7.0 (4.0)	7.0 (4.25)	7.0 (5.0)	0.020*
**Exercise duration** (min), median (IQR)	8.0 (4.0)	8.0 (5.0)	8.0 (4.0)	0.366
**Emotional status**				
Anger	162 (40.5)	75 (38.7)	87 (42.2)	0.467
Confusion	102 (25.5)	53 (27.3)	49 (23.8)	0.418
Depression	172 (43.0)	78 (40.2)	94 (45.6)	0.273
Fatigue	84 (21.0)	43 (22.2)	41 (19.9)	0.579
Tension	90 (22.5)	49 (25.3)	41 (19.9)	0.200
Vigor	24 (6.0)	10 (5.2)	14 (6.8)	0.490
Anxiety	106 (26.5)	53 (27.3)	53 (25.7)	0.719
Excitement	148 (37.0)	75 (38.7)	73 (35.4)	0.505
Happiness	146 (36.5)	75 (38.7)	71 (34.5)	0.384
Dejection	130 (32.5)	62 (32.0)	68 (33.2)	0.796

VO_2_max: maximal oxygen consumption. METs: metabolic equivalents of task. CDS-5: Cigarette Dependence Scale-5. IQR: interquartile range.

### Nicotine dependence and emotional status

The emotional status of nicotine-dependent participants was analyzed, and it was revealed that a significant proportion of them experienced negative emotions (Table 1), with depression (n=172; 43.0%) and anger (n=162; 40.5%) being the most prevalent. However, there were no significant differences in terms of emotional status between male and female participants (Table 1).

### Nicotine dependence and sport training behavior

The participants’ sport training behavior was analyzed using CBS-S, which revealed that the majority of participants scored from 3 to 5 in each module of sports training (Supplementary file Figure S2).

### Nicotine dependence and cardiopulmonary endurance

The cardiopulmonary endurance was determined by estimating VO_2_max, and the results revealed that the median VO_2_max level was 27.0 (IQR: 11.0), with no significant difference between male and female participants (Table 1). In addition, VO_2_max levels were significantly lower in all participants as well as in male and female participants with high nicotine dependence CDS-5 scores of 4 and 5 (Supplementary file Figures S3 A–C). In addition, Spearman’s correlation analysis revealed that nicotine dependence CDS-5 scores were negatively correlated with VO_2_max levels (r= -0.883, p<0.001) (Table 2 and Supplementary file Figure S3 D) in all participants as well as in males (r= -0.858, p<0.001) (Supplementary file Figure S3 E) and females (r= -0.904, p<0.001) (Table 2 and Supplementary file Figure S3 F). Furthermore, a stepwise linear regression analysis revealed that nicotine dependence CDS-5 scores were negatively related to VO_2_max levels, irrespective of age and gender (Table 2). In addition, the median treadmill METs value was 7.0 (IQR: 4.0), and the median exercise duration was 8.0 (IQR: 4.0) in all participants, with a significant difference in METS between male and female participants (p=0.020) (Table 1). Furthermore, Spearman’s correlation analysis revealed that nicotine dependence CDS-5 scores were negatively correlated with treadmill METs and exercise duration in all participants (r = -0.683, -0.689, respectively; all p<0.001) as well as in males (r= -0.662, -0.723, respectively; all p<0.001) and females (r= -0.686, -0.727, respectively; all p<0.001).

**Table 2:** Linear regression analysis of tobacco dependence in relation to age, physical activity level, and VO_2_ max

*Variable*	*Correlation analysis*		*Linear regression analysis*			
	*r*	*p*	*B*	*Standardized beta coefficient*	*t*	*p*
**Age** (years)	−0.011	0.831	0.002	0.003	0.155	0.877
**Gender**	−0.079	0.114	0.055	0.020	1.037	0.300
**Physical activity level**	−0.830	<0.001	−0.599	−0.344	−11.969	<0.001*
**VO_2_max**	−0.883	<0.001	−0.135	−0.643	−22.283	<0.001*

VO_2_max: maximal oxygen consumption. METs: metabolic equivalents of task.

* Statistically significant, p<0.001.

### Nicotine dependence and physical activity level

To determine whether physical activity affects nicotine dependence, Spearman’s correlation analysis revealed that nicotine dependence scores were negatively correlated with physical activity levels in all participants (r= -0.830, p<0.001) (Table 2 and Supplementary file Figure S4 A), as well as in males (r= -0.835, p<0.001) and females (r= -0.822, p<0.001). Furthermore, a stepwise linear regression analysis revealed that nicotine dependence scores were negatively related to physical activity levels in all participants, irrespective of age and gender (Table 2).

On the other hand, the impact of nicotine dependence on physical activity level was investigated. The physical activity level in the studied cohort was low in most participants (n=133; 33.2%) as well as in males (n=71; 36.6%) and females (n=62; 30.1%) (Table 1), with low physical activity being more prevalent in those with high nicotine dependence scores of 4 and 5 in all participants (χ^2^=365.2, p<0.001) (Supplementary file Figure S4 B), as well as in males (χ^2^=176.4, p<0.001) (Supplementary file Figure S4 C) and females (χ^2^=190.2, p<0.001) (Supplementary file Figure S4 D). Logistic regression analysis revealed that high nicotine dependence scores (odds ratio (AOR=14.66; 95% CI: 4.98–43.19, p<0.001) and low VO_2_max levels (AOR=0.71; 95% CI: 0.60–0.84, p<0.001) were independently related to low physical activity (Table 3), irrespective of age, gender, body weight, BMI, and alcohol consumption.

**Table 3:** Logistic regression analysis of physical activity level in relation to age, tobacco dependence, and VO_2_max

*Variable*	*Univariable analysis*		*Multivariable analysis*	
	*OR (95% CI)*	*p*	*AOR (95% CI)*	*p*
**Age** (years)	0.97 (0.85–1.05)	0.520	0.86 (0.75–0.99)	0.050
**Gender**	0.75 (0.49–1.13)	0.168	0.97 (0.42–2.25)	0.945
**BMI**	1.03 (0.97–1.10)	0.329	1.00 (0.87–1.16)	0.984
**Body weight** (kg)	1.02 (0.99–1.02)	0.181	0.99 (0.93–1.06)	0.848
**Alcohol consumption**	0.73 (0.47–1.13)	0.155	0.66 (0.34–1.30)	0.232
**Nicotine dependence (CDS-5 score)**	96.57 (40.20–231.99)	<0.001	14.66 (4.98–43.19)	<0.001*
**VO_2_max**	0.56 (0.49–0.64)	<0.001	0.71 (0.60–0.84)	<0.001*

AOR: adjusted odds ratio; adjusted for age, gender, BMI, body weight, and alcohol consumption. VO_2_max: maximal oxygen consumption. CDS-5: Cigarette Dependence Scale-5.

* Statistically significant, p<0.001.

To determine whether nicotine’s effect on physical activity level was mediated by affecting cardiopulmonary endurance, a mediation analysis was performed using the Sobel test, which revealed that altered cardiopulmonary endurance (VO_2_max) had no mediating role on the effect of nicotine addiction on lowering physical activity levels (z= -0.749, p=0.453).

## DISCUSSION

The study findings revealed that smoking is linked to negative emotions, particularly depression and anger. In addition, VO_2_max was inversely correlated with the extent of addiction to smoking, with lower VO_2_max levels associated with higher nicotine dependence. Furthermore, a significant decrease in physical activity level with increasing nicotine dependence was found, irrespective of age, gender, BMI, and alcohol consumption status.

Cigarette smoke contains over 4100 chemicals, including nicotine, which causes physical and psychological addiction. China has the highest tobacco consumption in the world. According to WHO reports, China has more than 300 million smokers, or nearly one-third of all smokers globally^17^. Unless adult men quit smoking widely, the annual number of tobacco-related deaths could rise from 1 million in 2010 to 3 million in 2050^18^. These data suggest that China’s tobacco control policies still need to be improved. The current state of smoking control in China is discouraging, particularly for young Chinese college students who smoke to form relationships with others and relieve stress and strain. However, it would endanger their physical health due to nicotine addiction. Therefore, it is important to implement efficient tobacco prevention programs and a smoking warning system for college students. A previous study reported that male college students who studied sports and medicine, came from non-single families with higher incomes, had psychological discomfort and lower learning efficacy, and lived in close proximity to others who were more likely to smoke^19^. Therefore, it is important to educate college students to deter them from smoking. College administrators and professors can help students avoid smoking more effectively by changing their attitudes and feelings toward learning.

The findings of this study revealed that smoking is linked to negative emotions, especially depression and anger. In this regard, the association between smoking and emotional and psychiatric disorders has been established in many studies. Data from surveys conducted in US and Australia from 2001–2007 revealed that adults with mental disorders smoked nearly twice as much as those without, and one-third of adult smokers had a 12-month mental disorder (31.7% in the US, and 32.4% in Australia), with female and younger smokers having higher rates of mental disorders^20^. Furthermore, smoking may contribute to the development and maintenance of anxiety disorders by modulating fear memory and emotion processing. A study of 1050 young adults in southeast Michigan revealed that females and males with nicotine addiction were more likely to develop alcohol and illicit substance disorders, anxiety disorders, and depression than non-dependent non-smokers and smokers^21^. In a systematic review of 47 population-based epidemiological studies, anxiety disorders were found to be a risk factor for smoking initiation and nicotine dependence, and cigarette smoking and nicotine dependence were risk factors for some anxiety disorders such as panic and generalized anxiety disorders^22^. In addition, the German Transitions in Alcohol Consumption and Smoking Study data of 1636 never smokers and 2437 ever smokers, revealed that nicotine-dependent smoking was significantly associated with various substance use, affective and anxiety disorders^23^. Furthermore, smoking increases the risk of bipolar disorder due to its effects on monoaminergic and glutamatergic systems, oxidative stress, and inflammatory and neurotrophic processes. Moreover, smoking was found to be associated with atrophy in several brain regions and smaller basal forebrain volume in elderly patients, compromising cholinergic reserve capacity and increasing the risk of dementia and Alzheimer disease^24^. These effects may be mediated by cardiovascular impairment from smoking and the cumulative direct cytotoxic effects of some cigarette smoke compounds^24^. On the other hand, people with mental illness are nearly twice as likely to smoke and less likely to quit. According to previous reports, smoking prevalence ranges from 44% to 88% in schizophrenia, 40% to 60% in major depression, and 55% to 70% in bipolar disorder^20^.

Smoking lowers cardiopulmonary endurance. It can cause coronary heart disease by promoting blood vessel plaque formation, which leads to arterial narrowing (atherosclerosis), lowering blood flow, and increasing the possibility of clotting. Furthermore, smoking can cause a number of respiratory and lung diseases, including emphysema, chronic obstructive pulmonary disease, and chronic bronchitis^20^. The cardiopulmonary exercise test and ergospirometry are two methods for assessing cardiorespiratory capacity. Maximum oxygen consumption is used to calculate cardiopulmonary functional capacity (VO_2_max), which represents the maximum capacity for oxygen absorption, transportation, and consumption. It has been reported that smoking reduces VO_2_max^7^. The present study revealed an inverse correlation between VO_2_max and the extent of addiction to smoking, with lower VO_2_max levels associated with higher nicotine dependence. Smoking reduces VO_2_max levels in several ways. First, tobacco smoke enters the bloodstream and binds to red blood cells, preventing oxygen from reaching the muscles and other body tissues. Second, there are two tobacco toxins that can harm people’s health: carbon monoxide and tar. Carbon monoxide replaces blood oxygen, depletes organ oxygen, and hinders organ function. Tar coats the lungs and impairs breathing^25^.

On the other hand, a significant decrease in physical activity level was found in this study with increasing addiction, which is consistent with previous studies on Korean non-elderly and elderly adults and Greek health science students^26^, indicating that smoking can negatively affect physical fitness. In addition, Su et al.^27^ found that current smoking was correlated with decreased physical fitness in tests of aerobic (3000 m run) and anaerobic (sit-ups and push-ups) capacity in 3669 Taiwanese military males. Similarly, smoking was associated with lower exercise levels and lower physical endurance – both cardiorespiratory (a 1.5 mile run) and muscular (sit-ups) – in 3045 Navy personnel^28^. In a systematic review of 50 articles, 60% of the articles reported a definite negative association between smoking and physical activity^29^. In a cross-sectional study of 2602 healthy adults in Tehran, smokers had an odds ratio of 4.88 (95% CI: 3.34–7.13) for having unsatisfactory physical activity compared to non-smokers^9^.

Smoking could affect physical activity in multiple ways. First, smoking lowers VO_2_max by deteriorating pulmonary conditions, which reduce oxygen delivery to the muscles. As a result, there is a rise in lactic acid, which causes fatigue, harder breathing, and more discomfort after exercise. Second, smoking consumes more energy because it uses more respiratory system muscles. Certainly, smoking is associated with a negative impact on muscle function, and it has been shown previously that muscle endurance is lower in smokers, as is exercise capacity in the absence of spirometric abnormalities or cardiac dysfunction^30^. Reductions in endurance have been shown to rapidly reverse within short periods of smoking cessation^31^.

In this regard, this study revealed that low VO_2_max levels were independently related to physical inactivity. However, a mediation analysis revealed that altered cardiopulmonary endurance (VO_2_max) had no mediating role on the effect of nicotine addiction on lowering physical activity levels. In contrast, a previous study found no correlation between VO_2_max and physical activity level, despite the fact that sedentary people consumed less oxygen than active people in general. Thus, more research on the effects of cardiopulmonary endurance and VO_2_max level on physical activity is required^32,33^. However, aerobic functions can be restored by quitting smoking and exercising regularly. In addition, the present study revealed that body weight, BMI, and alcohol consumption have no effect on physical activity levels. However, it was assumed that the findings reflect the profile of smokers, but cannot be attributed solely to smoking.

Interestingly, this study revealed that low physical activity was a risk factor for higher smoking dependence, indicating a bidirectional relationship between smoking and physical activity. Teenagers who engage in physical activity may also experience a decrease in harmful smoking behavior, with one additional week of physical activity leading to a 0.3% decline in the smoking probability and a 4.1% decrease in the number of cigarettes smoked in a month^34^. Thus, it is suggested that even modest increases in physical activity can improve health by reducing smoking.

### Strengths and limitations

There is much literature exploring the relationship between physical activity and smoking^29,35,36^. However, this is the first study to investigate the bidirectional effect of smoking on physical activity and the mechanism by which smoking could negatively affect physical activity by altering cardiopulmonary endurance. The findings will be useful in developing future tobacco addiction management strategies.

However, this study has some limitations. First, data were collected from participants via a questionnaire based on their responses, implying that response bias may exist. Second, because this study was limited to a single community, the findings cannot be generalized. Finally, it is necessary to investigate the impact of smoking cessation on cardiopulmonary endurance and physical activity level. However, this was not possible because all participants were current smokers.

## CONCLUSIONS

Tobacco smoking has a negative impact on emotional status. It also reduces cardiopulmonary endurance by reducing VO_2_max levels and negatively affects physical activity. Consequently, individuals with a higher tobacco addiction level tend to be less physically active. Accordingly, it is critical to implement effective tobacco prevention programs for college students, such as a smoking warning system and physical exercise training, as well as to educate them on how to quit smoking.

## Data Availability

The data supporting this research can be found in the Supplementary file.
