# Supplementary material for: Nicotine dependence affects cardiopulmonary endurance and physical activity in college students in Henan, China
Source: Tob Induc Dis. 2023 Jun 23;21:86. doi: 10.18332/tid/166133 (PMC10288961; doi:10.18332/tid/166133)
Supplement: Supplementary file 1 [file TID-21-86-s1.pdf]

Table S1. The Cigarette Dependence Scale-5

Questions	Response options	Recoding
1. Please rate your addition to cigarettes on a scale of 0 to 100: -I am Not addicted to cigarettes at all =0 -I am extremely addicted to cigarettes =100	Addiction	0-20=1 21-40 =2 41-60 =3 61-80=4 81-100=5
2. On average, how many cigarettes do you smoke per day?	Cigarettes/day	0-5=1 6-10=2 11-20=3 21-29=4
3. How soon after waking up do you usually smoke your first cigarette?	Minutes	0-5=5 6-15=4 16-30=3 31-60=2
4. For you, quitting smoking for good would be:	Impossible =5 Very difficult =4 Fairly difficult =3 Fairly easy =2	No recoding
5. After a few hours without smoking, I feel an irresistible urge to smoke	Totally disagree =1 Somewhat disagree =2 Neither agree nor disagree= 3 Somewhat agree =4 Fully agree = 5	

Supplementary Figure 1. Participants' flow chart.

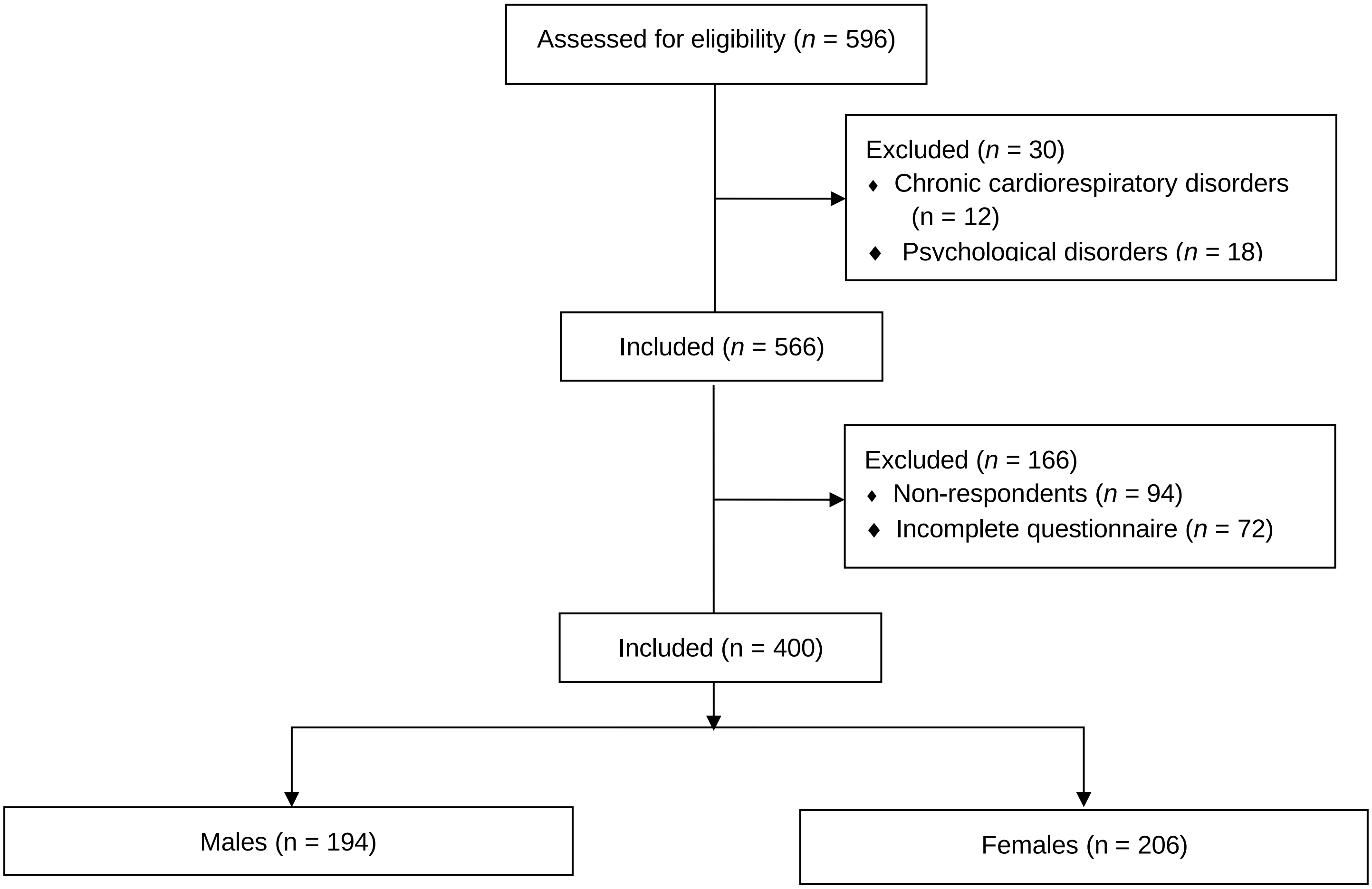

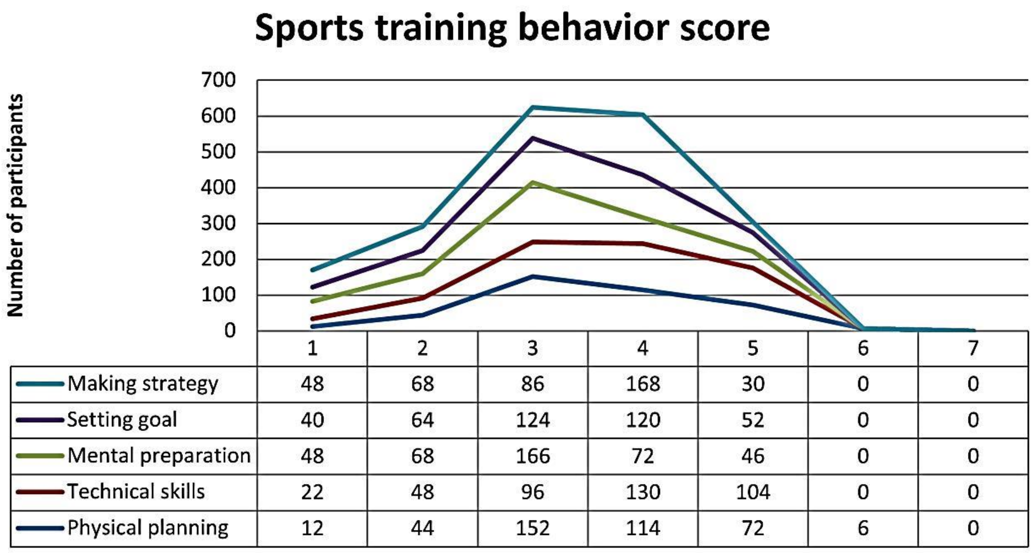

	1	2	3	4	5	6	7
Making strategy	48	68	86	168	30	0	0
Setting goal	40	64	124	120	52	0	0
Mental preparation	48	68	166	72	46	0	0
Technical skills	22	48	96	130	104	0	0
Physical planning	12	44	152	114	72	6	0

Supplementary Figure 2. Sports training behavior score using the Coaching Behaviour Scale for Sport (CBS-S) among the participants included in the study showing the number of participants in each score for each module.

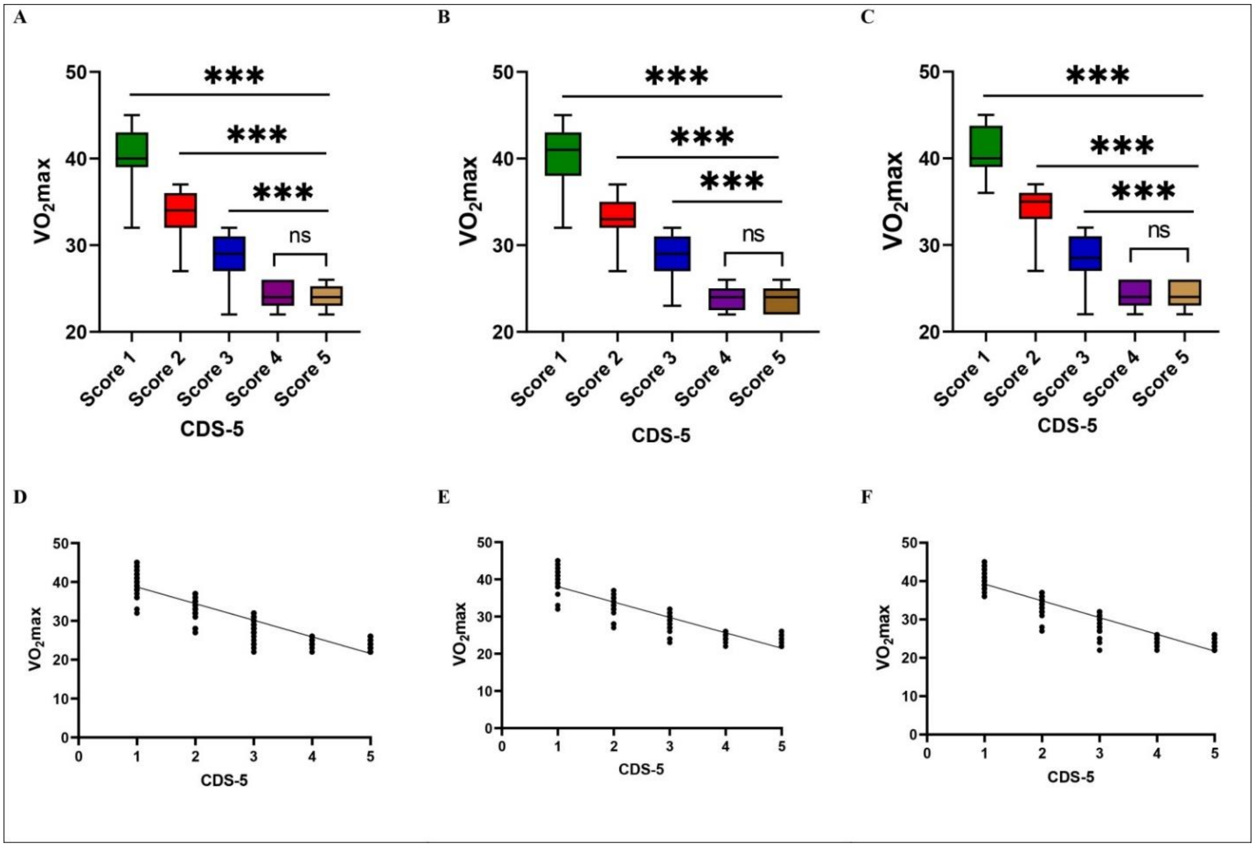

Supplementary Figure 3. The relationship between VO_2max levels and nicotine dependency CDS-5 scores in participants included in the study. (A) VO_2max levels among different CDS-5 scores in all participants (A), as well as male (B) and female (C) participants. Spearman's correlation analysis between VO_2max levels and CDS-5 scores in all participants (D), as well as male (E) and female (F) participants.

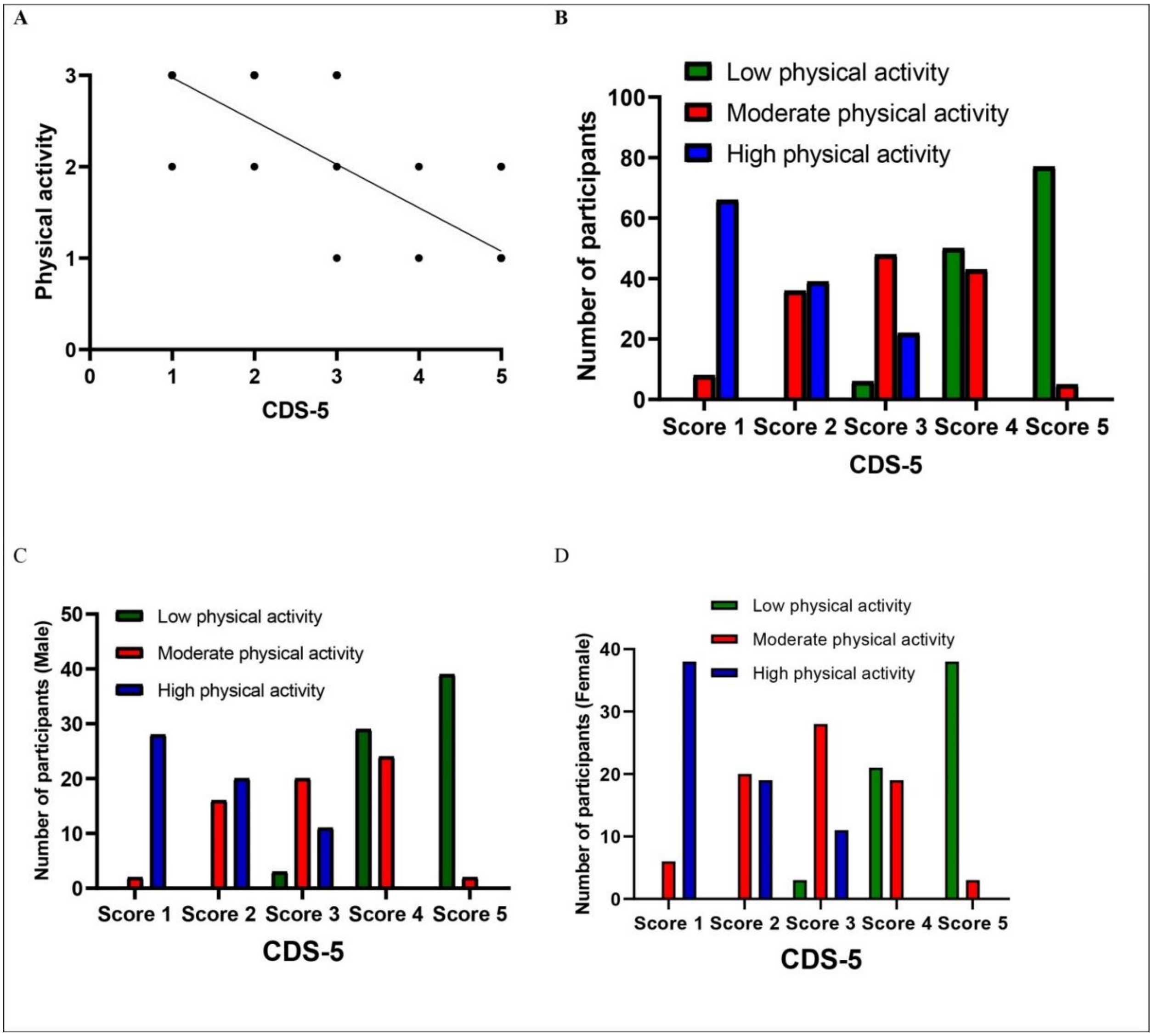

Supplementary Figure 4. The relationship between physical activity levels and nicotine dependency CDS-5 scores in participants included in the study. Spearman's Correlation analysis between physical activity levels and CDS-5 scores (A) and the number of all participants (B), male (C), and female (D) participants with different physical activity levels among different CDS-5 scores.

© 2023 Guo Y.
